# Improved YOLOv8-Based Method for the Carapace Keypoint Detection and Size Measurement of Chinese Mitten Crabs

**DOI:** 10.3390/ani15070941

**Published:** 2025-03-25

**Authors:** Ke Chen, Zhuquan Chen, Changbo Wang, Zhifan Zhou, Maohua Xiao, Hong Zhu, Dongfang Li, Weimin Liu

**Affiliations:** 1 College of Engineering, Nanjing Agricultural University, Nanjing 210031, China; ckyf@njau.edu.cn (K.C.); chenzhuquan@stu.njau.edu.cn (Z.C.); 9213011205@stu.njau.edu.cn (Z.Z.); dongfangli@njau.edu.cn (D.L.); 2 Kunshan Aquatic Technology Promotion Station, Kunshan 215300, China; dzxyjy2020@163.com; 3 Jiangsu Agricultural Machinery Development and Application Center, Nanjing 210017, China; 4 Jiangsu Three & Three Information Technology Company, Nanjing 210018, China; liuweimin@33iot.com

**Keywords:** Chinese mitten crab, carapace, YOLOv8l-pose, Swin Transformer, key point detection, size measurement

## Abstract

The carapace size of the Chinese mitten crab (*Eriocheir sinensis*) is a vital indicator for assessing the growth performance of crabs. However, measuring the carapace sizes of Chinese mitten crabs remains challenging due to environmental complexity, species-specific behavioral patterns, and the current limitations of data acquisition methods characterized by labor-intensive manual measurements and subjective empirical judgments. Our study proposes an automated carapace dimension-measuring method integrating enhanced computer vision techniques to address the above challenges. Specifically, we used the YOLOv8 algorithm combined with the pose keypoint detection algorithm to process Chinese mitten crab images to acquire carapace sizes. We redesigned the YOLOv8l-pose architecture by incorporating Swin Transformer as the backbone network to improve feature representation for multikeypoint detection on crab carapaces, significantly enhancing global contextual feature extraction capabilities. Furthermore, we refined the loss function to model spatial correlations between keypoint locations accurately and thus improve detection accuracy for dorsal carapace dimension key points in Chinese mitten crabs. Our system enabled noncontact size measurement to leverage the proportional relationship between precalibrated background markers and detected carapace keypoints. Experimental results demonstrated that our enhanced model achieved a mean average precision of 95.88%, representing a 2.61% improvement over the baseline. The overall object keypoint similarity reached 91.32%, with maximum and mean dimensional measurement errors of 4.8% and 2.34%, respectively, validating our method’s reliability for aquaculture applications.

## Simple Summary

The carapace dimensions of Chinese mitten crabs (*Eriocheir sinensis*) exhibit significant correlations with their growth stages, and precise measurement of these dimensions provides crucial support for aquaculture decision-making. To achieve automated carapace measurement, we developed an enhanced YOLOv8l-Pose algorithm that enables accurate detection of keypoints on crab carapaces. Subsequently, the acquired keypoints were connected and analyzed through background calibration markers combined with proportional relationships to establish a noncontact dimensional measurement system. Experimental results demonstrated maximum and average measurement errors of 4.8% and 2.34%, respectively, meeting practical requirements for aquaculture applications. This innovative approach provides effective technical support for intelligent crab farming by enabling the automated acquisition of growth parameters during cultivation processes.

## 1. Introduction

The Chinese mitten crab (*Eriocheir sinensis*), commonly known as the river crab, is a distinctive species in China and a vital component of the nation’s freshwater aquaculture, with an annual production nearing 900,000 tons [1]. However, crab farming relies heavily on farmers’ experience and manual feeding practices, often leading to disease outbreaks, uneven growth, feed waste, and deteriorating water quality [2]. As the industry expands, these traditional methods have proven to be inadequate for achieving precise feeding and management, making data-driven decision-making increasingly urgent [3]. A key element in optimizing aquaculture practices is the accurate assessment of crab growth by monitoring changes in growth rate, feed intake, and other critical parameters. Traditionally, this measurement involves manually capturing crabs from ponds to inspect shell sizes. This process is time-consuming, labor-intensive, and potentially harmful to the animals. Recent advances in computer vision offer a promising alternative. With its advantages of automation, high precision, and real-time processing, vision technology has emerged as an essential tool for aquatic product detection [4,5,6,7]. Utilizing this technology to identify underwater aquaculture targets is necessary for enhancing the intelligence and operational efficiency of farming equipment for Chinese mitten crabs [8].

Relying on acquiring images and building deep learning models provide a reliable solution to various complex scenarios [9]. By solving the problems of subtle classification features and overlapping masks, Gu et al. [10] developed R-TNET, a detection model tailored for the identification of precocious one-year-old crabs. Aiming to address the problems of the low detection accuracy and slow speed of detection algorithms for river crabs and bait in pond culture in underwater complex environments, Sun et al. [11] proposed a detection method for detecting river crabs and bait based on improved YOLOv5s. In the detection of the number and distribution of river crabs in ponds, Zhao et al. [12] used the optimized Retinex algorithm to enhance image contrast under the conditions of considerable underwater light attenuation and blurred fields of view and obtained improved recognition results. Meanwhile, Zhao et al. [13] employed EfficientNet-B0 extended with local linkage as the backbone network of a live crab detector, which could rapidly and accurately detect river crabs underwater, along with acquiring the statistics of the distribution of live crabs in ponds. Zhang et al. [14] also addressed the problem of recognizing river crabs underwater and proposed a lightweight river crab recognition model based on YOLOv5s for underwater environments; this model had better recognition accuracy and lighter weight than similar models. Fang et al. [15] proposed a lightweight deep learning model for YOLOv7-SPSD by integrating the Slimneck module, PConv, and the SimAM attention mechanism by using DLNA. The SimAM attention mechanism used the DepGraph pruning algorithm to remove redundant parameters to achieve model optimization. The model could accurately identify the junction of the tail and dorsal armor of river crabs and assisted in the automated dehulling of river crabs. Ji et al. [16] proposed a detection method for underwater river crab targets based on multiscale pyramid fusion image enhancement, image fusion, and a MobileCenterNet model. The detection method improved image enhancement and model feature extraction capability. However, the above study only aimed to identify different individual river crabs and detected the number, location, and specific parts of individual river crabs. It did not obtain the particular growth parameters of individual river crabs and could not measure the growing body size of river crabs, i.e., the measurement of the carapace size of river crabs.

Researchers have conducted studies on various farmed organisms to acquire specific growth information. Li et al. [17] proposed an automatic measurement method based on keypoint detection and monocular depth estimation using the YOLOv5s network for cattle, then employed keypoint detection by the Lite-HRNet network and extracted monocular depth information by applying the global–local path network to extract monocular depth information; this approach enabled the measurement of oblique body length, body height, chest depth, and hoof diameter of beef cattle under different distance and lighting conditions. Zhang et al. [18] proposed the noncontact measurement of sheep body dimensions based on machine vision in response to the limitations of current manual measurement methods. A unique data acquisition device was utilized to capture images at a fixed distance to measure sheep shoulder height, back height, hip height, and other body size data; the results showed that more than 90% of errors in the measurement of sheep body size were within 3%. Shi et al. [19] proposed a noncontact fish length estimation method using a stereovision system with LabVIEW algorithms to extract key measurement points at the tip of the muzzle and tail automatically; this method can estimate the length of a free-swimming fish with a high rate of accuracy and success. Through the construction of the YOLOv4-tiny model, Lai et al. [20] detected South American white shrimp in images and applied image processing algorithms for the background segmentation of the detected shrimp and estimation of shrimp body length; the proposed length estimation method had an average absolute error and average absolute relative error of 3.5 mm and 5.09%, respectively. In the above study, the target detection method was used to recognize cultured organisms from images, and the key dimensions of the cultured organisms were obtained through keypoint detection and image processing. However, river crabs are highly active, their cheliped gestures and directions vary, and stains and algae are often present on the surfaces of their shells. Therefore, applying existing methods to the real-time scenarios of the multipoint detection of river crab shells in the measurement of shell sizes is difficult, thus requiring detection models with substantial feature extraction capabilities.

Swin Transformer is a neural network architecture designed for computer vision tasks [21]. It achieves hierarchical representation by periodically moving windows with nonoverlapping localized windows. Each layer of Swin Transformer processes features in these small localized windows, and by changing the window positions in subsequent layers, the model can integrate information from broad regions of images. This window-shifting operation helps reduce computational complexity and allows for the efficient and scalable processing of images at high resolutions; therefore, Swin Transformer often outperforms previous state-of-the-art models while being more computationally efficient [22,23]. Moreover, combining Swin Transformer and YOLO architectures enables improved feature extraction for target detection tasks [24].

In summary, in this work, we propose an improved YOLOv8-based method for the keypoint detection and size measurement of river crab carapaces. First, data for river crabs are collected by building a data collection terminal. The Swin Transformer architecture is integrated into the YOLOv8l-pose model to improve the feature extraction capability and computational speed of the model and address the problem of the multipoint detection of river crab carapaces. The loss function of the YOLOv8l-pose model used for keypoint detection is improved such that it is in line with the actual situation of keypoint detection and accelerates the convergence speed of the model. The method breaks through the key technology for measuring river crab growth and body size, providing scientific decisions for river crab breeding and laying a technical foundation for the broad application of aquaculture intelligence.

## 2. Materials and Methods

At and before the juvenile stage, river crabs have tiny individual sizes, and the conditions for measurement are unavailable, resulting in the difficulty and necessity of measurement. Therefore, the primary study object is the river crab that had completed 13 molts and was stocked in adult crab ponds. River crabs, as aquatic crustaceans, select shelters for burrowing activities at different growth stages and are thus difficult to measure [25]. Moreover, the collection of growth data for underwater organisms is often delayed and unstable. In consideration of this situation, we collected data with an aquatic data collection terminal, which expands the function of crab trapping cages on the basis of the biological characteristics of river crabs themselves. These cages were placed during aquaculture, with the inlet positioned at the bottom of the water in the pond to allow river crabs to enter. The outlet at the end of a cage was lifted out of the water to connect with the image acquisition platform to collect images of river crabs through the image acquisition platform.

### 2.1. Overall Flow of the Proposed Method

**Figure 1:**
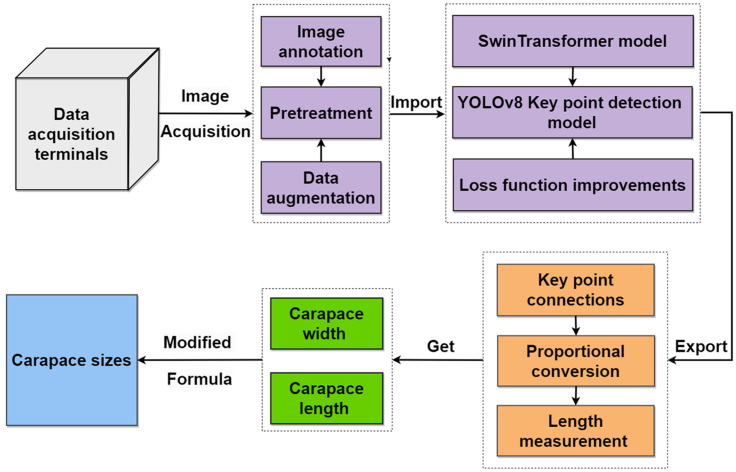
Overall methodology flow.

We propose a method for monitoring the growth condition of river crabs based on the improved YOLOv8l-pose algorithm. We obtained images and weights of river crabs by designing an aquatic platform as a data collection terminal and utilized the Swin Transformer architecture fused to the YOLOv8l-pose model. At the same time, we improved the loss function to make it in line with the keypoint detection scenario. The expected intersection over union (EIoU) loss function replaced the complete intersection over inion (CIoU) loss function. The keypoint loss function was introduced for river crab keypoint detection. Finally, the specified keypoints were connected to a line. Given that the size of the background plate was known, the distance between the two points, which is the required pixel size, was obtained through the scale formula. The overall methodology flow is shown in Figure 1.

### 2.2. Image Acquisition

The data acquisition terminal in this experiment was built in a river crab farm in Lishui District, Jiangsu Province. The shooting time of the collected images was selected as the afternoon when the natural light was sufficient, and the overwater platform with the ground cage was used to acquire the images. In the data acquisition terminal, the camera was set 30 cm away from the river crab, and the background was a 25 cm × 25 cm orange background that contrasted with the dark green color of the river crab itself, making the outlines of the collected river crabs clear and facilitating image processing.

**Figure 2:**
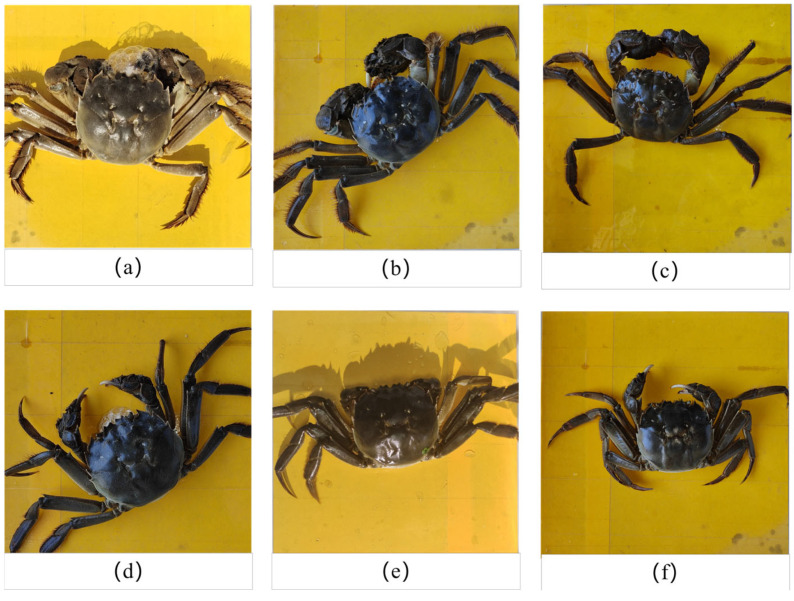
Acquired images. (**a**) Special grade male crab (≥250 g); (**b**) First class male crab (≥200 g); (**c**) Second class male crab (≥150 g); (**d**) Special grade female crab (≥225 g); (**e**) First class female crab (≥175 g); (**f**) Second class female crab (≥125 g).

Different sexes of river crabs have remarkable differences in dorsal armor width, dorsal armor length, and weight. In addition, river crabs of the same sex show minor differences when their growth conditions differ. By querying farmers, we found that river crabs on the market are divided into the special grade, first class, and second class in accordance with integrity degree, weight, and other factors. Hence, in selecting different grades of river crabs for filming, the ratio of male crabs to female crabs was approximately 1:1 to ensure the comprehensiveness of the overall dataset. The collection of images is shown in Figure 2. 

### 2.3. Preprocessing

#### 2.3.1. Image Enhancements

The number of collected images was 1256, and the overall sample dataset was small for deep learning. Therefore, data enhancement must be conducted on the original dataset to prevent overfitting and the poor generalization ability of the trained model and improve the overall model training effect. Data enhancement refers to processing original data in a certain way to expand the dataset without substantially increasing these data. Commonly used single-sample data enhancement methods are mainly divided into two categories: geometric and pixel transformation. Geometric transformation includes flipping, rotation, cropping, scaling, and panning. Pixel transformation includes noise removal, blurring, color transformation, and erasing. We used scale resizing, flipping, and color transformation to expand the original dataset to 5024 sheets.

#### 2.3.2. Dataset Labeling

**Figure 3:**
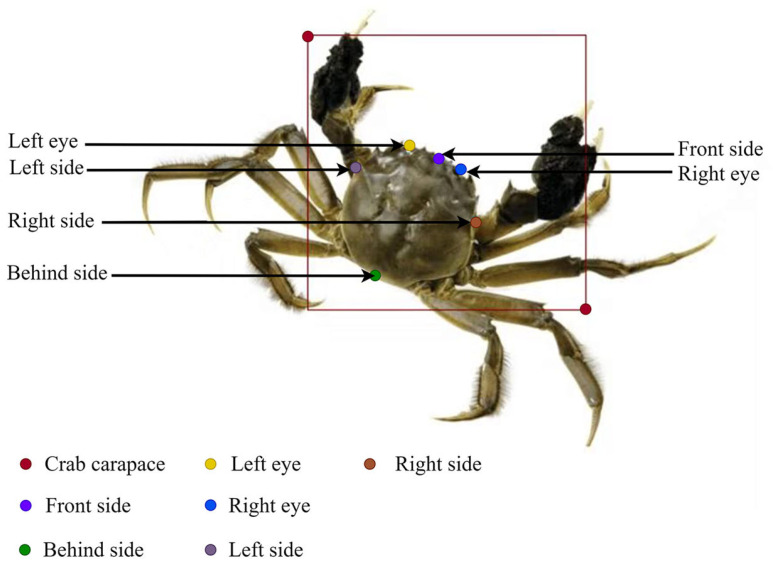
Keypoint labeling of river crabs.

All images had dimensions of 3000 × 4000 pixels. They were saved in JPG format, and river crabs were labeled using Labelme (v5.8.1), as shown in Figure 3. The specific process is as follows:

1. A rectangular box was used to frame the parts, including the double chelae and dorsal armor, of the river crab labeled as the carapace.
2. Six keypoints were used to label the left eye, right eye, and the front, back, left, and right sides of the river crab’s dorsal armor. Their corresponding English names were used as their labeling names, as shown in Table 1.
3. At the end of the labeling, a JSON file was automatically generated and converted into the txt file required by YOLOv8l.
4. The dataset is divided into training and validation sets in the ratio of 9:1, and the images in the training and validation sets numbered 4522 and 502 images, respectively.

### 2.4. YOLOv8 Improvements

#### 2.4.1. Model Structure

In this study, the YOLOv8l algorithm was applied to detect river crabs, where it performs target recognition and keypoint detection of the target parts. Its network structure is mainly composed of an input, a backbone, a neck, and a head [26]. The input adopts the method of stochastic data augmentation, which is used to scale, flip, and adjust image color, enriching the dataset while reducing the load on the GPU. The backbone consists of five convolutional modules, four C2f modules, and one SPPF module, which dramatically reduces the number of parameters required and has good feature extraction capability, reducing the computational burden. The neck adopts a feature pyramid network structure, which adjusts images with different resolutions by up- and down-sampling and enables further processing and feature fusion of the images to improve the generalization ability and robustness of the overall model. The head part adopts a decoupled head, which decomposes the original detection head and introduces the WIOU loss function, which dramatically improves the performance of the model and makes use of the features that have been extracted to make predictions of structures.

#### 2.4.2. Backbone Network Improvement

We introduced a modified model architecture that improves recognition speed and feature extraction capability to address the limitations of the original YOLOv8l in the real-time keypoint detection of river crab carapaces. Specifically, the YOLOv8l backbone was replaced with a Swin Transformer-based structure with few transformer blocks in each stage. This alteration considerably decreased the model’s computational parameters and workload while maintaining accuracy, reducing the demand for video memory and computational resources.

**Figure 4:**
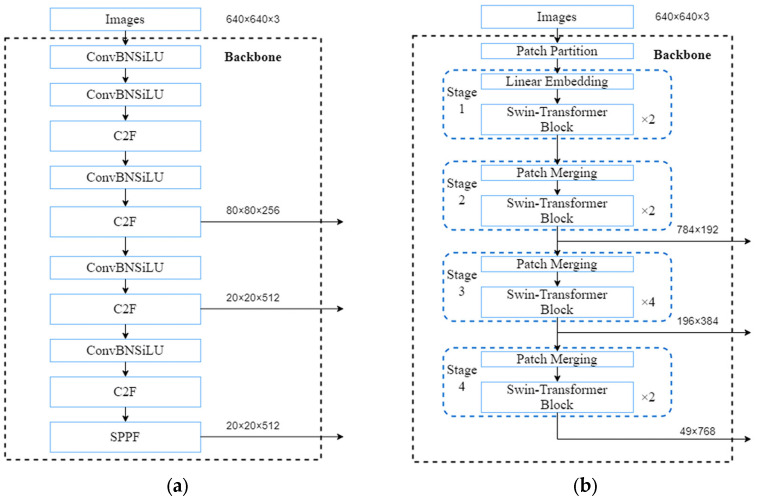
(**a**) Original backbone network. (**b**) Swin Transformer backbone network.

Furthermore, the Swin Transformer’s hierarchical attention mechanism partitions the input image or video into blocks, producing feature maps of varying sizes across different layers. By applying self-attention to each of these small feature maps, the model reduces training parameters and computational complexity and enhances recognition speed. The modified architecture is illustrated in Figure 4.

#### 2.4.3. Loss Function Improvement

The traditional YOLOv8 loss function comprises two parts: classification loss and bounding box regression loss. The latter typically includes the CIoU and distributional focal loss components. In this study, wherein the focus is on medium and large targets and the custom keypoints lie within the bounding box, enhancing bounding box accuracy is critical for reducing keypoint detection errors. Therefore, we replaced CIoU with EIoU and modified the aspect ratio factor to incorporate specific width-to-width and height-to-height losses, accelerating the model’s convergence. Additionally, we introduced object keypoint similarity (OKS) to develop a new keypoint loss function, ensuring that the overall loss reflects the practical requirements of keypoint detection with increased accuracy. The improved loss function is presented in Formula (1).

(1) $$L_{total}=\sum_{s,i,j,k,m}\left(\lambda_{1}L_{cls}+\lambda_{2}L_{EIoU}+\lambda_{3}L_{DFL}+\lambda_{4}L_{kepts}+\lambda_{5}L_{kepts_{\text{\_}conf}}\right),$$

where $L_{total}$ represents the total loss; $L_{cls}$ indicates the classification loss; $L_{EIoU}$ and $L_{DFL}$ denote the bounding box regression loss; $L_{kepts}$ signifies the loss of keypoints; $L_{kepts_{\text{\_}conf}}$ designates the loss of confidence in keypoints; $\lambda_{1}=0.5$, $\lambda_{2}=7.5$, $\lambda_{3}=1.5$, $\lambda_{4}=15$, and $\lambda_{5}=1.0$ are the weight parameters of each loss function.

The specific formulas are shown in Formulas (2)–(5):

(2) $$L_{EIoU}=L_{IoU}+L_{dis}+L_{asp},$$

(3) $$L_{IoU}=1-IoU,$$

(4) $$L_{dis}=\frac{\rho^{2}(b,b_{gt})}{c^{2}},$$

(5) $$L_{asp}=\frac{\rho^{2}(w,w_{gt})}{c_{w}^{2}}+\frac{\rho^{2}(h,h_{gt})}{c_{h}^{2}},$$

where IoU is mainly used to quantify the degree of overlap between the prediction and actual boxes and represents the ratio of the intersection and concatenation of the prediction and actual boxes. The value of IoU is in the range of [0, 1], and the larger it is, the more accurate the prediction, and the smaller it is, the more erroneous the prediction. Moreover, the higher the IoU threshold, the better the detection ability of the model. The specific calculation formula is shown in Formula (6):

(6) $$IoU=\frac{Area1\cap Area2}{Area1\cup Area2}.$$

**Figure 5:**
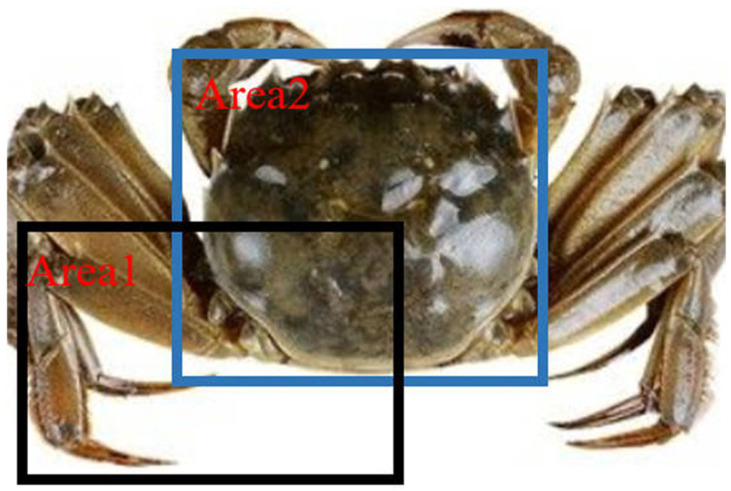
Actual IoU calculation chart for river crabs.

Area1 and Area2 in Formula (6) denote the area of the area of the prediction and real boxes, respectively, as shown in Figure 5.

In the above formula, $L_{IoU}$ is the IoU loss, $L_{dis}$ is the centroid loss, and $L_{asp}$ is the frame height loss. $b\;and\;b_{gt}$ are the centroids of the prediction and real frames, respectively. $\rho^{*}(\;)$ is used to find the distance between two points, and c is the diagonal distance between the prediction and real frames of the smallest external rectangle. w and $w_{gt}$ are the widths of the prediction and real frames, respectively, and $c_{w}$ is the width of the smallest external rectangle of the prediction box and the real frame of the two. h and $h_{gt}$ are the heights of the prediction box and real frame, respectively, and $c_{h}$ is the height of the minimum outer rectangle of the prediction and real boxes.

The specific formulas of $L_{kepts}$ are shown in Formulas (7) and (8):

(7) $$L_{kepts}=1-\sum_{n=1}^{N_{kepts}}OKS,$$

(8) $$OKS=\frac{\exp\left(d_{n}^{2}/2s^{2}k_{n}^{2}\right)\delta \left(v_{n}>0\right)}{\delta (v_{n}>0)},$$

where $d_{n}$ denotes the Euclidean distance between the predicted value of the nth keypoint and the manually labeled value, s denotes the square root of the area of the keypoint detection frame, $k_{n}$ denotes the keypoint-specific weights, and δ denotes the visibility flag of each keypoint.

### 2.5. Prediction of Crab Carapace Size Based on Keypoint Detection

#### 2.5.1. Keypoint Detection

Keypoint detection is an important basis for obtaining the growth condition of river crabs, and its result directly affects the accuracy of carapace size measurement. First, six keypoint detection targets were selected: the left eye, right eye, and the front, back, left, and right sides of the river crab’s dorsal armor.

The line segment formed by connecting the four keypoints of the anterior, posterior, left, and right sides of the dorsal armor of the river crab two by two was used as the preliminary width and length of the dorsal armor of the river crab. However, the high feature similarity of the four points mentioned above easily confuses and affects recognition results. Therefore, the most distinctive double eyes on the dorsal armor of river crabs were selected as the keypoints to determine the correct orientation of the dorsal armor and improve the accuracy of recognizing the keypoints of the dorsal armor of river crabs.

**Figure 6:**
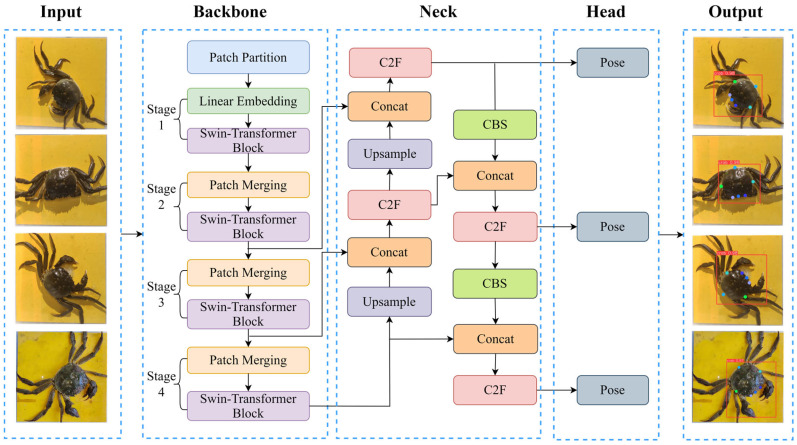
Keypoint detection for improved YOLOv8l modeling.

Second, after labeling the location and keypoints of the top view image of river crabs captured from the crab pond using Labelme software, the improved YOLOv8l algorithm was applied to locate the river crab target in the image and extract the keypoints of the phenotypic features of the river crab target and obtain the location of the keypoints of the left and right eyes of river crabs, as well as the front, back, left, and right sides of the dorsal armor of the crab in the crab image. Finally, the corresponding keypoints were connected to acquire the scale line segments of river crab armor by pixels. The keypoint detection process is shown in Figure 6.

#### 2.5.2. Prediction of Crab Carapace Size

In the acquisition of river crab images, a camera with a fixed shooting distance of 30 cm and a fixed angle directly above the camera was used to take pictures, and accurate dorsal armor planar dimensions were obtained by simple calibration on a 25 cm × 25 cm base plate. However, linear regression must be used to fit an equation that corrects the effect of river crab thickness by collecting a large amount of pixel-size measured data on dorsal armor size. During measurement, only the pixel width/length of the dorsal armor was detected. The specific formula below (9) can calculate the actual dorsal armor size with increased accuracy:

(9) $$d_{real}=a\cdot d_{pixel}+b,$$

where $d_{real}$ is the real physical size of the dorsal armor of river crabs (e.g., width/length); $d_{pixel}$ is the pixel distance obtained from keypoint detection or contour measurement; a and b are the regression coefficients obtained by fitting a large number of samples.

## 3. Results and Discussion

### 3.1. Experimental Environment

The hardware and software systems and parameters that we used in this experiment are as follows: Windows 10 Operating System, CPU AMD Ryzen9 5950X16-Creo Processor, RAM 128G, GPU NVIDIA GeForce RTX 3090, and Development Environment Python3.8 and Pytorch1.10.1

### 3.2. Evaluation Indicators

Mean average precision (mAP), precision, and recall are applied as evaluation metrics to evaluate the performance of the model for river crab target detection. Moreover, OKS is employed to evaluate the performance of keypoint detection.

#### 3.2.1. Precision and Recall

Precision and recall are commonly used metrics in the context of object and target detection. The precision rate indicates the proportion of the number of positive samples in the predicted samples to the number of all positive samples, and the recall rate indicates the proportion of the actual number of positive samples in the predicted samples to the number of all samples. In target detection, if the bounding box predicted by the model coincides with the actual bounding box, then the prediction is considered correct. However, if the real bounding box coincides with the predicted bounding box, then the sample is deemed correctly recalled. The specific calculation formulas for precision and recall are shown in Formulas (10) and (11), respectively:

(10) $$Precision=\frac{TP}{TP+FP}\times 100\%,$$

(11) $$Recall=\frac{TP}{TP+FN}\times 100\%.$$

#### 3.2.2. mAP

mAP is a vital evaluation metric in target detection to assess the detection accuracy of the model in each category. It is calculated by sorting each category in accordance with the predicted confidence level, and the area enclosed under the P–R curve is called AP. mAP is the average area enclosed by all categories. The higher its value, the better the detection performance of the model. Its specific calculation formula is shown in Formula (12):

(12) $$mAP=\frac{\sum_{c=1}^{c}AP(c)}{c}$$

#### 3.2.3. OKS

OKS is a metric that is commonly used to evaluate the results of object keypoint detection. It quantifies the similarity between the keypoints detected and actual keypoints and measures the model’s performance by considering the spatial relationship. The specific formula is shown in Formula (8) above.

### 3.3. Results Showcase

Both the baseline model and the enhanced model were trained on the pre-established dataset, with subsequent comparative analysis of their performance discrepancies across multiple metrics to demonstrate experimental outcomes.

#### 3.3.1. Precision and Recall Evaluation

**Figure 7:**
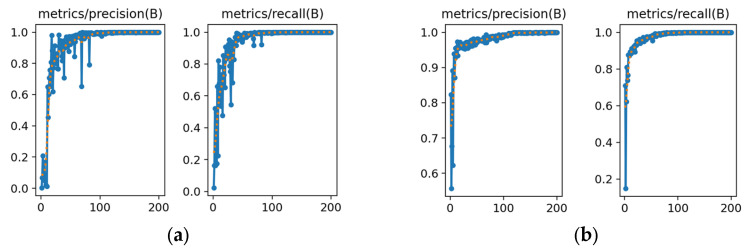
(**a**) Precision and recall of the original YOLOv8l model. (**b**) Precision and recall of the improved YOLOv8l Model.

The resulting model precision vs. recall curves are shown below:

As shown in Figure 7, the precision and recall curves obtained from the improved YOLOv8l model concerning the number of training times have fewer fluctuations and a gentler trend than those from the original model, indicating that the improved model provides better performance at lower training times than the original model.

#### 3.3.2. mAP Evaluation

**Figure 8:**
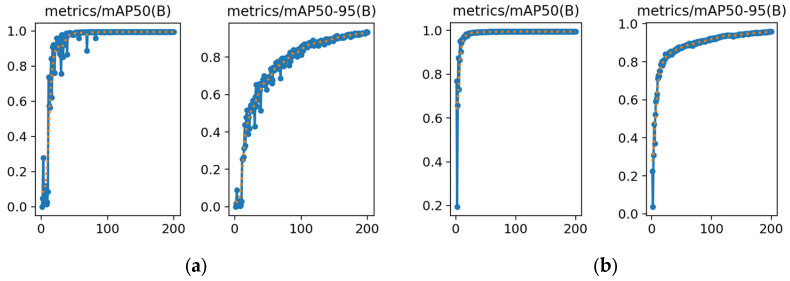
(**a**) Detection accuracy of the original YOLOv8l model. (**b**) Detection accuracy of the improved YOLOv8l model.

The final resulting model mAP is shown below:

The mAP training results are presented in Figure 8, and the overall mAP is high because the purpose of this model is river crab recognition and detection. mAP50 indicates an average accuracy at an IoU threshold of 0.5, whereas mAP50–95 indicates accuracy at IoU thresholds of 50–95. mAP50–95 calculates then averages mAP values over a range of IoU state values of 50–95%, enabling a highly accurate assessment of the model’s performance at different IoU thresholds. It is, therefore, comprehensive and applicable to this study. The improved YOLOv8l model epochs to 100 when the mAP reaches more than 91%, and its final mAP is 95.88%, indicating that it performs better than the original model.

#### 3.3.3. Training Loss

**Figure 9:**
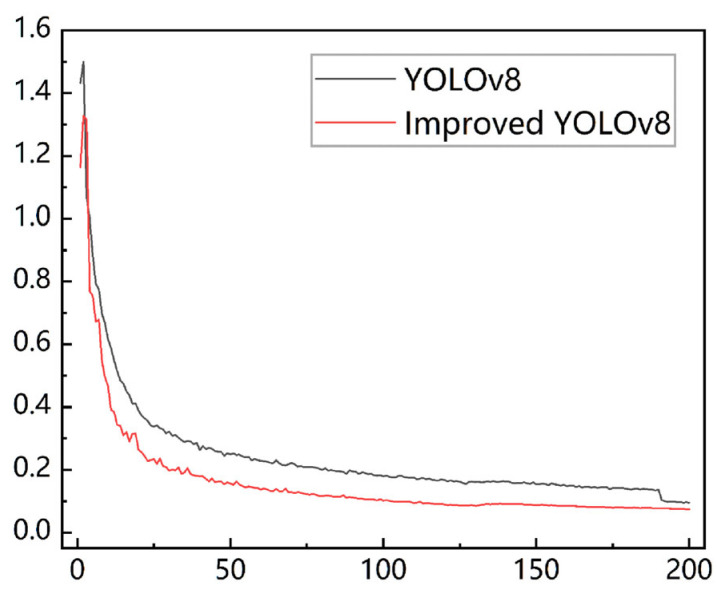
Original model training loss vs. improved model training loss.

The obtained final loss curves are shown in Figure 9, which shows that with the increase in training times, each model loss gradually decreases and converges to a stable state. Comparison with the training loss of the original model shows that the improved loss function can promote the model to learn the probability distribution of the target location quickly and improve the prediction results of keypoint detection, effectively improving the convergence speed of the model and the accuracy of target detection.

#### 3.3.4. Keypoint Detection Results

**Figure 10:**
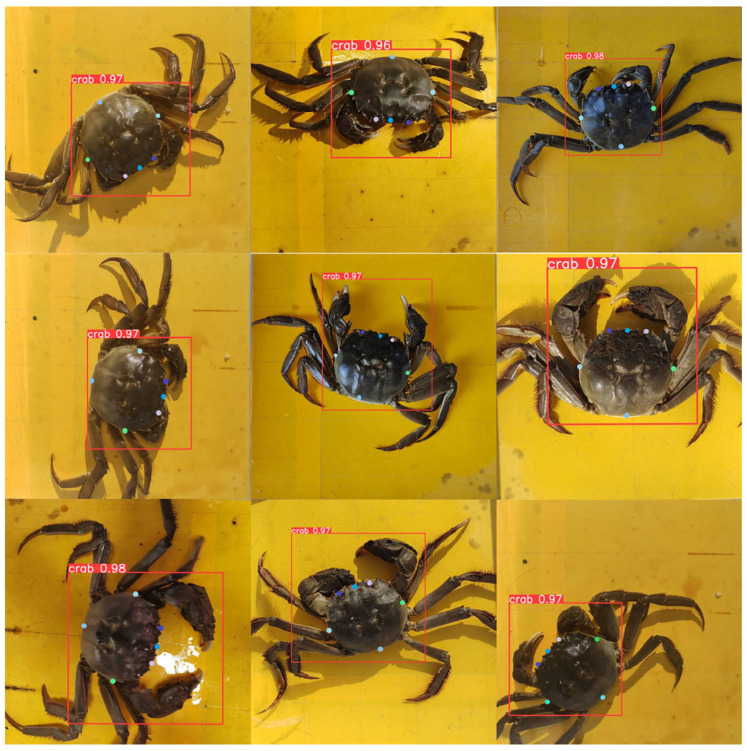
Model test results.

After we trained the model with 4522 images, we employed the improved YOLOv8l algorithm to extract the target and keypoint information of river crabs. The visualization results are provided in Figure 10, where the red boxes represent the target detection of river crabs, and colored dots indicate keypoints. The results demonstrate that the model suits river crabs with varying postures and growth stages, exhibiting good robustness and generalization ability.

**Figure 11:**
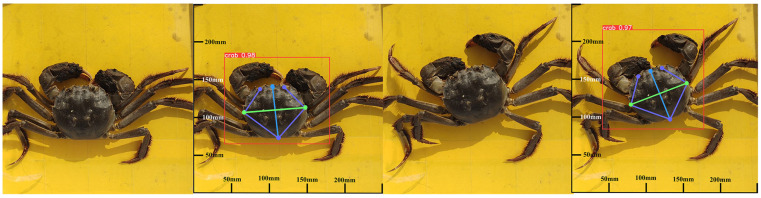
Keypoint connection and size prediction through model test results.

Keypoint connection and size prediction through the model test results are shown in Figure 11, where the purple lines connect the keypoints to outline the carapace contour, and green and light blue lines denote the width and length of the carapace. Meanwhile, a Cartesian coordinate system was established with the origin at the bottom-left corner of the image. The coordinates of each keypoint and their average OKS were recorded. Points with OKS < 0.5 were excluded from the display. The remaining visible keypoints closely approximate a normal distribution. The mean and standard deviation of OKS values for identical keypoints across multiple images were calculated to validate keypoint recognition accuracy (Table 2). The results demonstrate high reliability, with an overall mean OKS of 91.32% for morphological keypoint detection, indicating robust confidence levels and minimal variability.

### 3.4. Size Prediction Experiment

**Figure 12:**
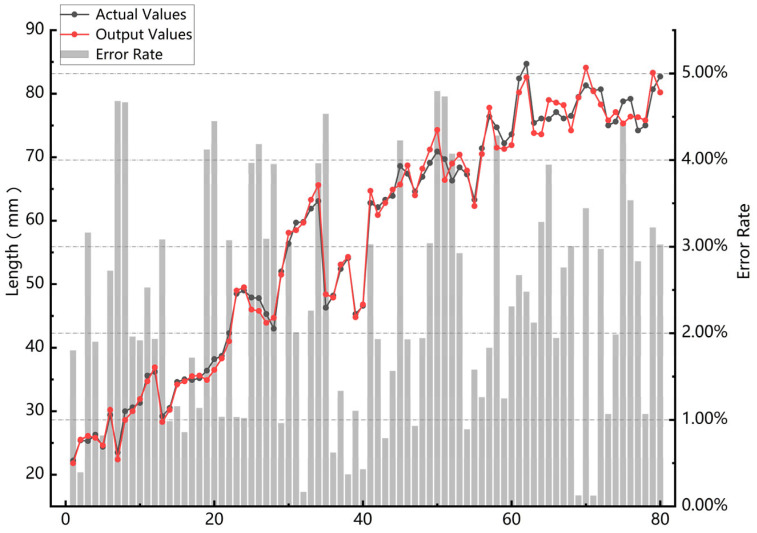
Experimental results of size prediction.

Given that the size of the shooting background plate is known to be 25 mm × 25 mm, the scale formula can be used to derive the distance between two points as the size of the requested pixel. A total of 40 river crab samples, with 10 different growth stages and weight specifications according to the data collection terminal, were used. The length and width of their dorsal armor were measured. The size estimated by the model was compared with the actual measured size to calculate the error, as shown in Figure 12. The maximum error obtained is 4.8%. The average absolute error is 2.34%, which could be attributed to the effect of the keypoint’s size and positional error, but it is within acceptable limits.

## 4. Conclusions

In this study, a method for measuring the carapace sizes of river crabs was designed. It acquired the images of river crabs through a data acquisition terminal. It adopted the improved YOLOv8l model for keypoint detection to extract the image information of river crabs and complete the measurement of the carapace sizes of river crabs. The main conclusions obtained in this work are as follows:

(1) In consideration of the unique growth characteristics of river crabs, a data acquisition terminal was built to collect images of river crabs. A large number of images of river crab carapace sizes were collected and labeled, and data enhancement was conducted to expand the dataset.
(2) YOLOv8l was improved by introducing the Swin Transformer module into the backbone network. At the same time, the loss function in the YOLOv8l model was improved. The improved YOLOv8l model was enhanced in terms of accuracy, recall, AP, and convergence speed. Specifically, the AP of target detection was 95.88%, and the total average of OKS for keypoint detection was 91.32%.
(3) The improved YOLOv8l model was utilized for keypoint detection. The length proportionality between the background plate and keypoints of river crabs was utilized to determine the actual size and value of river crabs. The final test verified that the average absolute error of size measurement data was 2.34%, which met the test requirements.
(4) The method for the measurement of the shell sizes of river crabs proposed in this study enabled the fast and accurate nondestructive measurement of the growth condition of river crabs. It can collect key growth information of river crabs, provide exact data support for intelligent farming, and assist farmers in their farming decisions. However, there are limitations in creating application software based on the proposed model, and the proposed model needs to be combined with a data acquisition terminal that is set to the proposed specific image-capturing conditions to be applied.

## Figures and Tables

**Table 1:** Keypoint labeling of river crabs.

Keypoints	Labeling Tags	Specific Labeling Location
Left eye	left_eye	Crab’s left eye eyeball
Right eye	right_eye	Crab’s right eye eyeball
Left side	left_side	Last spine of crab dorsal armor, left side, posteriorly.
Right side	right_side	Last spine of crab dorsal armor, right side, posteriorly.
Front side	front_side	Crab between two spines at the center point between the eyes.
Behind side	behind_side	Corresponding points on the posterior side of the crab on the axis of the anterior side of the dorsal armor

**Table 2:** Mean and standard deviation statistics of the similarity of target keypoints.

Keypoints	Anterior Side	Posterior Side	Left Eye	Right Eye	Left Side	Right Side
Mean	0.932	0.937	0.896	0.912	0.883	0.919
Standard deviation	0.030	0.014	0.046	0.039	0.049	0.032

## Data Availability

The datasets presented in this article are not readily available because the data are part of an ongoing study. Requests to access the datasets should be directed to chenzhuquan@stu.njau.edu.cn.

## Abbreviations

The following abbreviations are used in this manuscript:

mAP	Mean average precision
OKS	Object keypoint similarity
EIoU	Expected intersection over union
CIoU	Complete intersection over union
